# Genomic evaluation by including dominance effects and inbreeding depression for purebred and crossbred performance with an application in pigs

**DOI:** 10.1186/s12711-016-0271-4

**Published:** 2016-11-25

**Authors:** Tao Xiang, Ole Fredslund Christensen, Zulma Gladis Vitezica, Andres Legarra

**Affiliations:** 1 Department of Molecular Biology and Genetics, Center for Quantitative Genetics and Genomics, Aarhus University, 8830 Tjele, Denmark; 2 UR1388 GenPhySE, INRA, CS-52627, 31326 Castanet-Tolosan, France; 3 INP, ENSAT, GenPhySE, Université de Toulouse, 31326 Castanet-Tolosan, France

## Abstract

**Background:**

Improved performance of crossbred animals is partly due to heterosis. One of the major genetic bases of heterosis is dominance, but it is seldom used in pedigree-based genetic evaluation of livestock. Recently, a trivariate genomic best linear unbiased prediction (GBLUP) model including dominance was developed, which can distinguish purebreds from crossbred animals explicitly. The objectives of this study were: (1) methodological, to show that inclusion of marker-based inbreeding accounts for directional dominance and inbreeding depression in purebred and crossbred animals, to revisit variance components of additive and dominance genetic effects using this model, and to develop marker-based estimators of genetic correlations between purebred and crossbred animals and of correlations of allele substitution effects between breeds; (2) to evaluate the impact of accounting for dominance effects and inbreeding depression on predictive ability for total number of piglets born (TNB) in a pig dataset composed of two purebred populations and their crossbreds. We also developed an equivalent model that makes the estimation of variance components tractable.

**Results:**

For TNB in Danish Landrace and Yorkshire populations and their reciprocal crosses, the estimated proportions of dominance genetic variance to additive genetic variance ranged from 5 to 11%. Genetic correlations between breeding values for purebred and crossbred performances for TNB ranged from 0.79 to 0.95 for Landrace and from 0.43 to 0.54 for Yorkshire across models. The estimated correlation of allele substitution effects between Landrace and Yorkshire was low for purebred performances, but high for crossbred performances. Predictive ability for crossbred animals was similar with or without dominance. The inbreeding depression effect increased predictive ability and the estimated inbreeding depression parameter was more negative for Landrace than for Yorkshire animals and was in between for crossbred animals.

**Conclusions:**

Methodological developments led to closed-form estimators of inbreeding depression, variance components and correlations that can be easily interpreted in a quantitative genetics context. Our results confirm that genetic correlations of breeding values between purebred and crossbred performances within breed are positive and moderate. Inclusion of dominance in the GBLUP model does not improve predictive ability for crossbred animals, whereas inclusion of inbreeding depression does.

## Background

Crossbreeding is primarily and intensively applied in meat production systems [1], especially for swine and poultry. Crossbreeding capitalizes on heterosis effects and complementarity between breeds, and results in an increased performance of crossbred animals compared to purebred animals [1]. In terminal crossbreeding systems, selection on purebred animals to maximize their crossbred performance is the ultimate goal [2, 3]. Due to the existence of genotype-by-environment interaction effects and non-additive genetic effects in combination with different allele frequencies in different breeds [3, 4], the genetic correlation of breeding values between purebred and crossbred performances ($r_{PC}$) is usually lower than 1 [1, 5], and therefore, purebred performance under nucleus conditions may not be an optimal predictor for crossbred performance in commercial animals [4, 6].

One of the major genetic bases of heterosis is dominance [7, 8]. At the level of gene action, dominance is due to interactions between alleles at the same locus [9]. In pedigree-based genetic evaluation, dominance is rarely included because large-scale datasets that comprise a high proportion of full sibs are required to obtain accurate estimates and because the computational complexity is high [10]. With the recent availability of single nucleotide polymorphism (SNP) information and the development of genomic selection, estimation of the dominance effects of SNPs has become more feasible [11, 12].

Genomic evaluation has been successfully used in purebred [13, 14] and crossbred populations [15–17]. However, these studies generally ignore the dominance effects. A number of studies have been carried out on genomic evaluation including dominance effects using either simulated [18] or real purebred data [9, 12].

Recently, several studies [19, 20] have tried to extend genomic evaluation including dominance effects from purebred performance to crossbred performance. However, they either used genomic information on purebred animals only [19] or applied a genomic model that assumed that all animals belong to a single population, and thus the variance components were estimated based only on the genotyped crossbred animals [20]. Nevertheless, combining purebred and crossbred information is essential to implement genetic evaluation for crossbred performance [1, 19]. Furthermore, because of genotype-by-environment interaction effects and different patterns of linkage disequilibrium (LD) between SNPs and quantitative trait loci (QTL), the effects of SNPs may be breed-specific [21]. To overcome these issues, a trivariate genomic best linear unbiased predictor (GBLUP) model that explicitly distinguishes between purebred and crossbred data and includes dominance was recently developed by Vitezica et al. [22]. This model allowed the estimation of different, yet correlated, additive and dominance marker effects in crossbred and purebred individuals. However, the empirical predictive ability of the trivariate GBLUP model has not been evaluated yet.

Thus, the current study had the following objectives: (1) to show how genomic inbreeding can be meaningfully included in GBLUP, even for crossbred animals; (2) to estimate the variance components of additive and dominance genetic effects by using data on total number of piglets born (TNB) in two Danish purebred and one crossbred pig populations using the trivariate GBLUP model; (3) to show how to derive, from variance component estimates, estimated genetic correlations of breeding values between purebred and crossbred performances in each pure breed, and also correlations of allele substitution effects between the two pure breeds; and (4) to evaluate the impact of dominance effects from genomic information on genomic evaluation by comparing accuracies of estimated genomic values in different cross-validation scenarios.

## Methods

### Animals and genotypes

We begin this section with a short presentation of the data used in the study, with the aim of defining the notation for the methodological developments that follow. For this study, all datasets were provided by the Danish Pig Research Centre. Data from three Danish pig populations were analyzed simultaneously: Landrace (L), Yorkshire (Y) and their reciprocal crosses (LY). Only data on TNB data for the first parity of sows in the three populations were used. In total, there were 2126, 2218 and 5143 genotyped sows with own records on TNB for L, Y and LY, respectively. Instead of using original records, corrected phenotypic values of TNB were used as dependent variables for the trivariate GBLUP model, because the pre-correction for non-genetic effects, such as herd-year-season, month at farrowing, and service sire was more accurately achieved on a larger dataset (293,339 L, 180,112 Y, and 10,974 LY). Among the crossbred animals, 7407 LY had a Landrace sire and a Yorkshire dam, while 3567 LY had a Yorkshire dam and a Landrace sire; L and Y populations were from nucleus farms and LY from a commercial farm. The litters of purebred sows were both purebred and crossbred litters. The relationship between LY-L and LY-Y are comparable since, in both cases, parents of the F1 animals are in the purebred datasets; further details about the model used for the pre-correction are in [17]. All the purebred sows had first farrowing dates between 2003 and 2013, while the crossbred sows first farrowed between 2010 and 2013. Only five of these purebred L and Y sows were dams of the LY.

The pedigrees for both purebred and crossbred sows were available and all crossbred animals were traced back to their purebred ancestors until 1994 by the DMU Trace program [23], as was done for the larger dataset used for pre-correction. Consequently, 8227 L, 9851 Y and 5143 LY individuals were in the pedigree. The dataset of pre-corrected TNB records for genotyped individuals is termed “full genomic dataset” throughout the whole paper, and it should not be confused with the larger dataset used to do the pre-correction.

For the “full genomic dataset”, purebred sows were genotyped with the Illumina PorcineSNP60 Genotyping BeadChip [24], while the crossbred sows were genotyped with a 8.5 K GGP-Porcine Low Density Illumina Bead SNP chip [25]. SNP quality controls (such as: call rate for individuals ≥80%; call rate for SNPs ≥90%; minor allele frequencies ≥0.01; etc.) were applied on the same dataset in a previous study [26], which provides more details. Then, for the crossbred individuals, imputation from low density to moderate density was done by using a joint reference panel of the two pure breeds [26] using the software Beagle version 3.3.2 [27] (imputation accuracies ≥95% in terms of correlation coefficients and ≥99% in terms of correct rates between imputed and true genotypes). Finally, 41,009 SNPs were available for all the recorded purebred and crossbred sows.

### Considering genomic inbreeding and heterosis

Inbreeding can be defined as the proportion of homozygous SNPs across all loci for each animal, as suggested by several authors (e.g., [28]). If there is directional dominance causing inbreeding depression [29], then inbreeding should be considered in the genetic evaluation models [30]. Otherwise, using pedigree or marker data, estimates of genetic parameters are inflated [30, 31]. In Vitezica et al. [22], genomic inbreeding was fitted as a covariate and, in the current study, we prove this reasoning by using a parametric genomic model, such as a GBLUP.

Theory and evidence of directional dominance (equivalently, inbreeding depression) suggest that dominance effects of genes (here associated to markers) should have a priori a positive value for traits that exhibit inbreeding depression or heterosis. If we call **d** the vector of dominance marker effects, the following prior distribution is plausible:

$$({\mathbf{d}})\sim\,N\left( {{\mathbf{1}}\mu_{d} ,{\mathbf{I}}\sigma_{d}^{2} } \right),$$

where *μ*_*d*_ is the overall mean of dominance effects, which should be positive if there is heterosis due to dominance. A typical model for genomic prediction is that in Toro and Varona [11]:

1 $${\mathbf{y}} = {\mathbf{X}} {\boldsymbol{\upbeta}} + {\mathbf{Za}} + {\mathbf{Wd}} + {\mathbf{e}},$$

where **y** contains phenotypic values; **Xβ** stands for fixed effects and random effects other than additive and dominance effects; **a** is the vector of “biological” additive SNP effects, **d** is the vector of “biological” dominance SNP effects for each of the markers; matrix **Z** has entries 1, 0, −1, for SNP genotypes AA, Aa and aa, respectively, while matrix **W** has entries 0, 1, 0 for SNP genotypes AA, Aa and aa, respectively. **e** is the vector of overall random residual effects.

Typically, genetic models require **a** and **d** to have zero means, which is not true for **d** when directional dominance exist. Defining ${\mathbf{d}}^{*} = {\mathbf{d}} - {\text{E(}}{\mathbf{d}} )$, then ${\text{E}}\left( {{\mathbf{d}}^{*} } \right) = {\mathbf{0}}$, and Eq. (1) can be written as:

$$\begin{aligned} {\mathbf{y}} & = {\mathbf{X}}{\boldsymbol{\upbeta}} + {\mathbf{Za}} + {\mathbf{W}}\left( {{\mathbf{d}}^{*} + {\mathbf{E}}({\mathbf{d}})} \right) + {\mathbf{e}} \\ & = {\mathbf{X}}{\boldsymbol{\upbeta}} + {\mathbf{Za}} + {\mathbf{Wd}}^{*} + {\mathbf{W1}}\mu_{d} + {\mathbf{e}}. \\ \end{aligned}$$

The term ${\mathbf{W1}}\mu_{d}$ is actually an average of dominance effects for each individual and is equal to ${\mathbf{h}}\mu_{d} ,$ where **h** = **W1** contains the row-sums of **W**, i.e. individual heterozygosities (it should be noted that **W** has a value of 1 at heterozygous loci for an individual). Inbreeding coefficients **f** can be calculated as:

$${\mathbf{f}} = {\mathbf{1}}- {\mathbf{h}}/N,$$

where *N* is the number of SNPs. Then, the prior means ${\mathbf{h}}\mu_{d}$ can be rewritten as:

$${\mathbf{h}}\mu_{d} = \left( {{\mathbf{1}} - {\mathbf{f}}} \right)N\mu_{d} = {\mathbf{1}}N\mu_{d} + {\mathbf{f}}\left( { - N\mu_{d} } \right).$$

The term ${\mathbf{1}}N\mu_{d}$ is confounded with the overall mean of the model (**μ**), while the term ${\mathbf{f}}\left( { - N\mu_{d} } \right)$ models the inbreeding depression and $b = \left( { - N\mu_{d} } \right)$ is the inbreeding depression parameter summed over the SNPs, which has to be estimated. Thus, the linear model including genomic inbreeding is, finally:

$${\mathbf{y}} = {\mathbf{X}}{\boldsymbol{\upbeta}} + {\mathbf{f}}b + {\mathbf{Za}} + {\mathbf{Wd}}^{ *} + {\mathbf{e}}.$$

Thus, we have proven why fitting overall homozygosity for the individual as a measure of inbreeding depression accounts for directional dominance.

### Estimating genetic (co)variances of markers with additive and dominance effects

A trivariate model based on “biological” (genotypic) additive and dominance effects of SNPs [22, 32], and including genomic inbreeding as above, was applied considering TNB as a different trait in each population:

2 $$\begin{aligned} {\mathbf{y}}_{L} & = {\mathbf{1}}\mu_{L} + {\mathbf{f}}_{L} b_{L} + {\mathbf{Z}}_{L} {\mathbf{a}}_{L} + {\mathbf{W}}_{L} {\mathbf{d}}_{L} + {\mathbf{e}}_{L} , \\ {\mathbf{y}}_{Y} & = {\mathbf{1}}\mu_{Y} + {\mathbf{f}}_{Y} b_{Y} + {\mathbf{Z}}_{Y} {\mathbf{a}}_{Y} + {\mathbf{W}}_{Y} {\mathbf{d}}_{Y} + {\mathbf{e}}_{Y} , \\ {\mathbf{y}}_{LY} & = {\mathbf{1}}\mu_{LY} + {\mathbf{f}}_{Y} b_{Y} + {\mathbf{Z}}_{LY} {\mathbf{a}}_{LY} + {\mathbf{W}}_{LY} {\mathbf{d}}_{LY} + {\mathbf{e}}_{LY} , \\ \end{aligned}$$

where ${\mathbf{y}}_{L} ,$ ${\mathbf{y}}_{Y}$ and ${\mathbf{y}}_{LY}$ contain corrected phenotypic values for purebred L, purebred Y and crossbred LY sows, respectively; $\mu_{L} ,$ $\mu_{Y}$ and $\mu_{LY}$ are the respective means; ${\mathbf{a}}_{L}$, ${\mathbf{a}}_{Y}$ and ${\mathbf{a}}_{LY}$ are the “biological” additive SNP effects and ${\mathbf{d}}_{L}$, ${\mathbf{d}}_{Y}$ and ${\mathbf{d}}_{LY}$ are the “biological” dominance SNP effects for each of the SNPs for L, Y and LY, respectively; matrices **Z** and **W** are as above; ${\mathbf{f}}_{L} b_{L}$, ${\mathbf{f}}_{Y} b_{Y}$ and ${\mathbf{f}}_{LY} b_{LY}$ model the inbreeding depression for L, Y and LY populations; ${\mathbf{e}}_{L}$, ${\mathbf{e}}_{Y}$ and ${\mathbf{e}}_{LY}$ are the overall random residual effects.

Note that “biological” is used here to refer to the genotypic additive and dominance values of the SNPs, to distinguish them from the traditional treatment of quantitative genetics in terms of “statistical” effects (breeding values and dominance deviations) [32].

The above equations can be reformulated to genotypic values of individuals instead of SNPs, in order to be compatible with the classical GBLUP model and animal breeding software, such as BLUPF90 [33] and DMU [34]:

3 $$\begin{aligned} {\mathbf{y}}_{L} & = {\mathbf{1}}\mu_{L} + {\mathbf{f}}_{L} b_{L} + {\mathbf{u}}_{L} + {\mathbf{v}}_{L} + {\mathbf{e}}_{L} , \\ {\mathbf{y}}_{Y} & = {\mathbf{1}}\mu_{Y} + {\mathbf{f}}_{Y} b_{Y} + {\mathbf{u}}_{Y} + {\mathbf{v}}_{Y} + {\mathbf{e}}_{Y} , \\ {\mathbf{y}}_{LY} & = {\mathbf{1}}\mu_{LY} + {\mathbf{f}}_{LY} b_{LY} + {\mathbf{u}}_{LY} + {\mathbf{v}}_{LY} + {\mathbf{e}}_{LY} . \\ \end{aligned}$$

Note that **u** and **v** are vectors of genotypic additive and dominance effects and therefore cannot be directly compared to breeding values and dominance deviations in the pedigree-based genetic evaluation. In addition, **f** is a vector of genomic inbreeding coefficients and *b* is a population-specific inbreeding depression parameter per unit of genomic inbreeding, respectively. Note that there is potentially inbreeding depression at the level of the crossbred animals, although, first, the numeric values of the vector **f** should be smaller since crossbred animals have a higher level of heterozygosity, and second, the estimates of the inbreeding depression parameters (*b*) do not need to be identical across the three populations, which thus gives considerable flexibility.

In terms of the genotypic additive effects **u**, the variances within each breed are:

$$\begin{aligned} Var\left( {{\mathbf{u}}_{L} } \right) & = var\left( {{\mathbf{Z}}_{L} {\mathbf{a}}_{L} } \right) = {\mathbf{Z}}_{L} {\mathbf{Z}}_{L}^{\varvec{'}} \sigma_{{a_{L} }}^{2} , \\ Var\left( {{\mathbf{u}}_{Y} } \right) & = var\left( {{\mathbf{Z}}_{Y} {\mathbf{a}}_{Y} } \right) = {\mathbf{Z}}_{Y} {\mathbf{Z}}_{Y}^{\varvec{'}} \sigma_{{a_{Y} }}^{2} , \\ Var\left( {{\mathbf{u}}_{LY} } \right) & = var\left( {{\mathbf{Z}}_{LY} {\mathbf{a}}_{LY} } \right) = {\mathbf{Z}}_{LY} {\mathbf{Z}}_{LY}^{\varvec{'}} \sigma_{{a_{LY} }}^{2} , \\ \end{aligned}$$

where $\sigma_{{a_{L} }}^{2}$, $\sigma_{{a_{Y} }}^{2}$ and $\sigma_{{a_{LY} }}^{2}$ are the additive variances of SNP effects in breeds L, Y and LY, respectively. The covariances between the genotypic additive effects **u** are:

4 $$Cov\left( {\begin{array}{*{20}c} {{\mathbf{u}}_{L} } \\ {{\mathbf{u}}_{Y} } \\ {{\mathbf{u}}_{LY} } \\ \end{array} } \right) = \left( {\begin{array}{*{20}c} {{\mathbf{Z}}_{L} {\mathbf{Z}}_{L}^{\varvec{'}} \sigma_{{a_{L} }}^{2} } & {{\mathbf{Z}}_{L} {\mathbf{Z}}_{Y}^{\varvec{'}} \sigma_{{a_{L,Y} }} } & {{\mathbf{Z}}_{L} {\mathbf{Z}}_{LY}^{\varvec{'}} \sigma_{{a_{L,LY} }} } \\ {{\mathbf{Z}}_{Y} {\mathbf{Z}}_{L}^{\varvec{'}} \sigma_{{a_{L,Y} }} } & {{\mathbf{Z}}_{Y} {\mathbf{Z}}_{Y}^{\varvec{'}} \sigma_{{a_{Y} }}^{2} } & {{\mathbf{Z}}_{Y} {\mathbf{Z}}_{LY}^{\varvec{'}} \sigma_{{a_{Y,LY} }} } \\ {{\mathbf{Z}}_{LY} {\mathbf{Z}}_{L}^{\varvec{'}} \sigma_{{a_{L,LY} }} } & {{\mathbf{Z}}_{LY} {\mathbf{Z}}_{Y}^{\varvec{'}} \sigma_{{a_{Y,LY} }} } & {{\mathbf{Z}}_{LY} {\mathbf{Z}}_{LY}^{\varvec{'}} \sigma_{{a_{LY} }}^{2} } \\ \end{array} } \right),$$

where $\sigma_{{a_{L,Y} }}$, $\sigma_{{a_{L,LY} }}$ and $\sigma_{{a_{Y,LY} }}$ are the additive covariances of SNP effects between populations L and Y, populations L and LY, and populations Y and LY, respectively. Analogous structures exist for dominance genotypic effects:

$$Cov\left( {\begin{array}{*{20}c} {{\mathbf{v}}_{L} } \\ {{\mathbf{v}}_{Y} } \\ {{\mathbf{v}}_{LY} } \\ \end{array} } \right) = \left( {\begin{array}{*{20}c} {{\mathbf{W}}_{L} {\mathbf{W}}_{L}^{\varvec{'}} \sigma_{{d_{L} }}^{2} } & {{\mathbf{W}}_{L} {\mathbf{W}}_{Y}^{\varvec{'}} \sigma_{{d_{L,Y} }} } & {{\mathbf{W}}_{L} {\mathbf{W}}_{LY}^{\varvec{'}} \sigma_{{d_{L,LY} }} } \\ {{\mathbf{W}}_{Y} {\mathbf{W}}_{L}^{\varvec{'}} \sigma_{{d_{L,Y} }} } & {{\mathbf{W}}_{Y} {\mathbf{W}}_{Y}^{\varvec{'}} \sigma_{{d_{Y} }}^{2} } & {{\mathbf{W}}_{Y} {\mathbf{W}}_{LY}^{\varvec{'}} \sigma_{{d_{Y,LY} }} } \\ {{\mathbf{W}}_{LY} {\mathbf{W}}_{L}^{\varvec{'}} \sigma_{{d_{L,LY} }} } & {{\mathbf{W}}_{LY} {\mathbf{W}}_{Y}^{\varvec{'}} \sigma_{{d_{Y,LY} }} } & {{\mathbf{W}}_{LY} {\mathbf{W}}_{LY}^{\varvec{'}} \sigma_{{d_{LY} }}^{2} } \\ \end{array} } \right).$$

### Estimation of marker-based variance components using an equivalent model

The variance components $\sigma_{{a_{L} }}^{2} ,$ $\sigma_{{a_{Y} }}^{2} ,$ $\sigma_{{a_{LY} }}^{2}$ and $\sigma_{{a_{L,Y} }} ,$ $\sigma_{{a_{L,LY} }} ,$ $\sigma_{{a_{Y,LY} }}$ in Eq. (4) cannot be estimated by regular methods or software (i.e. REML or Gibbs sampling) because they cannot be factorized out from Eq. (4). To fit such a multivariate structure, we used an equivalent model. Additional effects need to be defined, even if they are of no interest per se. For instance, the vectors of hypothetical genotypic additive effects of the genotypes of the L breed on the scale of breed Y (${\mathbf{u}}_{L,Y}$) and LY (${\mathbf{u}}_{L,LY}$) have variance–covariance matrices ${\mathbf{Z}}_{\text{L}} {\mathbf{Z}}_{\text{L}}^{'}\upsigma_{{{\text{a}}_{\text{Y}} }}^{2}$ and ${\mathbf{Z}}_{\text{L}} {\mathbf{Z}}_{\text{L}}^{'}\upsigma_{{{\text{a}}_{\text{LY}} }}^{2}$, respectively. Thus, as a whole, the genetic variance and covariance structure for the genotypic additive effects **u** are:

$$\begin{aligned} Var({\mathbf{u}}) & = {\text{var}}\left[ {\begin{array}{*{20}c} {{\mathbf{u}}_{L} } \\ {{\mathbf{u}}_{L,Y} } \\ {{\mathbf{u}}_{L,LY} } \\ {{\mathbf{u}}_{Y,L} } \\ {{\mathbf{u}}_{Y} } \\ {{\mathbf{u}}_{Y,LY} } \\ {{\mathbf{u}}_{LY,L} } \\ {{\mathbf{u}}_{LY,Y} } \\ {{\mathbf{u}}_{LY} } \\ \end{array} } \right] = {\text{var}}\left[ {\begin{array}{*{20}c} {{\mathbf{Z}}_{L} {\mathbf{a}}_{L} } \\ {{\mathbf{Z}}_{L} {\mathbf{a}}_{Y} } \\ {{\mathbf{Z}}_{L} {\mathbf{a}}_{LY} } \\ {{\mathbf{Z}}_{Y} {\mathbf{a}}_{L} } \\ {{\mathbf{Z}}_{Y} {\mathbf{a}}_{Y} } \\ {{\mathbf{Z}}_{Y} {\mathbf{a}}_{LY} } \\ {{\mathbf{Z}}_{LY} {\mathbf{a}}_{L} } \\ {{\mathbf{Z}}_{LY} {\mathbf{a}}_{Y} } \\ {{\mathbf{Z}}_{LY} {\mathbf{a}}_{LY} } \\ \end{array} } \right] \\ & = \left[ {\begin{array}{*{20}c} {{\mathbf{Z}}_{L} {\mathbf{Z}}_{L}^{\varvec{'}} } & {{\mathbf{Z}}_{L} {\mathbf{Z}}_{Y}^{\varvec{'}} } & {{\mathbf{Z}}_{L} {\mathbf{Z}}_{LY}^{\varvec{'}} } \\ {{\mathbf{Z}}_{Y} {\mathbf{Z}}_{{L}}^{\varvec{'}} } & {{\mathbf{Z}}_{Y} {\mathbf{Z}}_{Y}^{\varvec{'}} } & {{\mathbf{Z}}_{Y} {\mathbf{Z}}_{LY}^{\varvec{'}} } \\ {{\mathbf{Z}}_{LY} {\mathbf{Z}}_{L}^{\varvec{'}} } & {{\mathbf{Z}}_{LY} {\mathbf{Z}}_{Y}^{\varvec{'}} } & {{\mathbf{Z}}_{LY} {\mathbf{Z}}_{LY}^{\varvec{'}} } \\ \end{array} } \right] \otimes \left[ {\begin{array}{*{20}c} {\sigma_{{a_{L} }}^{2} } & {\sigma_{{a_{L,Y} }} } & {\sigma_{{a_{L,LY} }} } \\ {\sigma_{{a_{Y,L} }} } & {\sigma_{{a_{Y} }}^{2} } & {\sigma_{{a_{Y,LY} }} } \\ {\sigma_{{a_{LY,L} }} } & {\sigma_{{a_{LY,Y} }} } & {\sigma_{{a_{LY} }}^{2} } \\ \end{array} } \right] \\ & = {\mathbf{ZZ}}^{\varvec{'}} \otimes \left[ {\begin{array}{*{20}c} {\sigma_{{a_{L} }}^{2} } & {\sigma_{{a_{L,Y} }} } & {\sigma_{{a_{L,LY} }} } \\ {\sigma_{{a_{Y,L} }} } & {\sigma_{{a_{Y} }}^{2} } & {\sigma_{{a_{Y,LY} }} } \\ {\sigma_{{a_{LY,L} }} } & {\sigma_{{a_{LY,Y} }} } & {\sigma_{{a_{LY} }}^{2} } \\ \end{array} } \right] , \\ \end{aligned}$$

where matrix **Z** contains elements 1, 0, −1 for the three genotypes, and is defined across the three breeds, $${\mathbf{Z}} = \left[ {\begin{array}{*{20}c} {{\mathbf{Z}}_{L} } \\ {{\mathbf{Z}}_{Y} } \\ {{\mathbf{Z}}_{LY} } \\ \end{array} } \right]$$.

To construct a relationship matrix similar to the classical **G**-matrix of GBLUP [35], Vitezica et al. [22] introduced a normalized genomic relationship matrix ${\mathbf{G}} = \frac{{{\mathbf{ZZ}}^{\prime } }}{{\left\{ {{\text{tr}}\left[ {{\mathbf{ZZ}}^{\prime } } \right]} \right\}/{\text{n}}}}$, where n is the number of animals across the three populations and the division by $\left\{ {{\text{tr}}\left[ {{\mathbf{ZZ}}^{\prime } } \right]} \right\}/{\text{n}}$ scales the matrix such that the average of the diagonal elements equals 1. This alters the variances across genotypic additive effects **u** in the following way:

5 $$\begin{aligned} Var({\mathbf{u}}) & = var\left[ {\begin{array}{*{20}c} {{\mathbf{u}}_{L} } \\ {{\mathbf{u}}_{L,Y} } \\ {{\mathbf{u}}_{L,LY} } \\ {{\mathbf{u}}_{Y,L} } \\ {{\mathbf{u}}_{Y} } \\ {{\mathbf{u}}_{Y,LY} } \\ {{\mathbf{u}}_{LY,L} } \\ {{\mathbf{u}}_{LY,Y} } \\ {{\mathbf{u}}_{LY} } \\ \end{array} } \right] = {\mathbf{ZZ}}^{{\prime }} \otimes \left[ {\begin{array}{*{20}c} {\sigma_{{a_{L} }}^{2} } & {\sigma_{{a_{L,Y} }} } & {\sigma_{{a_{L,LY} }} } \\ {\sigma_{{a_{Y,L} }} } & {\sigma_{{a_{Y} }}^{2} } & {\sigma_{{a_{Y,LY} }} } \\ {\sigma_{{a_{LY,L} }} } & {\sigma_{{a_{LY,Y} }} } & {\sigma_{{a_{LY} }}^{2} } \\ \end{array} } \right] \\ & = \left( {{\mathbf{G}} \times \frac{{\left\{ {{\text{tr}}\left[ {{\mathbf{ZZ}}^{{\prime }} } \right]} \right\}}}{\text{n}}} \right) \otimes \left[ {\begin{array}{*{20}c} {\sigma_{{a_{L} }}^{2} } & {\sigma_{{a_{L,Y} }} } & {\sigma_{{a_{L,LY} }} } \\ {\sigma_{{a_{Y,L} }} } & {\sigma_{{a_{Y} }}^{2} } & {\sigma_{{a_{Y,LY} }} } \\ {\sigma_{{a_{LY,L} }} } & {\sigma_{{a_{LY,Y} }} } & {\sigma_{{a_{LY} }}^{2} } \\ \end{array} } \right] \\ & = {\mathbf{G}}\varvec{ } \otimes \left[ {\begin{array}{*{20}c} {\sigma_{{A_{L} }}^{2} } & {\sigma_{{A_{L} A_{Y} }} } & {\sigma_{{A_{L} A_{LY} }} } \\ {\sigma_{{A_{Y} A_{L} }} } & {\sigma_{{A_{Y} }}^{2} } & {\sigma_{{A_{Y} A_{LY} }} } \\ {\sigma_{{A_{L} A_{LY} }} } & {\sigma_{{A_{Y} A_{LY} }} } & {\sigma_{{A_{LY} }}^{2} } \\ \end{array} } \right] = {\mathbf{G}} \otimes {\mathbf{G}}_{0} , \\ \end{aligned}$$

where **G**_0_ are variance components associated to the genotypic additive effects **u**. This structure (a Kronecker product) is compatible with animal breeding software for BLUP and REML and the variance–covariance component **G**_0_ can be estimated in a straightforward manner. Then, the (co)variances of additive genotypic effects of SNPs across populations can be obtained as:

6 $$\begin{aligned} \left[ {\begin{array}{*{20}c} {\sigma_{{a_{L} }}^{2} } & {\sigma_{{a_{L,Y} }} } & {\sigma_{{a_{L,LY} }} } \\ {\sigma_{{a_{Y,L} }} } & {\sigma_{{a_{Y} }}^{2} } & {\sigma_{{a_{Y,LY} }} } \\ {\sigma_{{a_{LY,L} }} } & {\sigma_{{a_{LY,Y} }} } & {\sigma_{{a_{LY} }}^{2} } \\ \end{array} } \right] & = {\mathbf{G}}_{0}/\left\{ {{\text{tr}}\left[ {{\mathbf{ZZ}}^{\prime } } \right]} \right\}/{\text{n}} \\ & = \left[ {\begin{array}{*{20}c} {\sigma_{{A_{L} }}^{2} } & {\sigma_{{A_{L} A_{Y} }} } & {\sigma_{{A_{L} A_{LY} }} } \\ {\sigma_{{A_{Y} A_{L} }} } & {\sigma_{{A_{Y} }}^{2} } & {\sigma_{{A_{Y} A_{LY} }} } \\ {\sigma_{{A_{L} A_{LY} }} } & {\sigma_{{A_{Y} A_{LY} }} } & {\sigma_{{A_{LY} }}^{2} } \\ \end{array} } \right] /\left\{ {{\text{tr}}\left[ {{\mathbf{ZZ}}^{\prime } } \right]} \right\}/{\text{n}} .\\ \end{aligned}$$

The variances across genotypic dominance effects **v** are altered in a similar way:

7 $$\begin{aligned} Var({\mathbf{v}}) & = var\left[ {\begin{array}{*{20}c} {{\mathbf{v}}_{L} } \\ {{\mathbf{v}}_{L,Y} } \\ {{\mathbf{v}}_{L,LY} } \\ {{\mathbf{v}}_{Y,L} } \\ {{\mathbf{v}}_{Y} } \\ {{\mathbf{v}}_{Y,LY} } \\ {{\mathbf{v}}_{LY,L} } \\ {{\mathbf{v}}_{LY,Y} } \\ {{\mathbf{v}}_{LY} } \\ \end{array} } \right] \\ & = {\mathbf{D}}\varvec{ } \otimes \left[ {\begin{array}{*{20}c} {\sigma_{{D_{L} }}^{2} } & {\sigma_{{D_{L} D_{Y} }} } & {\sigma_{{D_{L} D_{LY} }} } \\ {\sigma_{{D_{Y} D_{L} }} } & {\sigma_{{D_{Y} }}^{2} } & {\sigma_{{D_{Y} D_{LY} }} } \\ {\sigma_{{D_{L} D_{LY} }} } & {\sigma_{{D_{Y} D_{LY} }} } & {\sigma_{{D_{LY} }}^{2} } \\ \end{array} } \right] = {\mathbf{D}}\varvec{ } \otimes {\mathbf{D}}_{0} , \\ \end{aligned}$$

where **D**_0_ contains variances and covariances associated to the genotypic dominance effects **v** and ${\mathbf{D}} = \frac{{{\mathbf{WW}}^{\prime } }}{{\left\{ {{\text{tr}}\left[ {{\mathbf{WW}}^{\prime } } \right]} \right\}/{\text{n}}}},$ where the matrix **W** contains elements 0, 1, 0 for the three genotypes, and is defined across the three breeds $$\left( {\mathbf{W}} = \left[ {\begin{array}{*{20}c} {{\mathbf{W}}_{L} } \\ {{\mathbf{W}}_{Y} } \\ {{\mathbf{W}}_{LY} } \\ \end{array} } \right]\right)$$ and $${\mathbf{W}}^{\prime } = \left[ {\begin{array}{*{20}c} {{\mathbf{W}}_{L}^{\varvec{'}} } & {{\mathbf{W}}_{Y}^{\varvec{'}} } & {{\mathbf{W}}_{LY}^{\varvec{'}} } \\ \end{array} } \right]$$. Then, the (co)variances of dominance genotypic effects of SNPs are:

8 $$\begin{aligned} \left[ {\begin{array}{*{20}c} {\sigma_{{d_{L} }}^{2} } & {\sigma_{{d_{L,Y} }} } & {\sigma_{{d_{L,LY} }} } \\ {\sigma_{{d_{Y,L} }} } & {\sigma_{{d_{Y} }}^{2} } & {\sigma_{{d_{Y,LY} }} } \\ {\sigma_{{d_{LY,L} }} } & {\sigma_{{d_{LY,Y} }} } & {\sigma_{{d_{LY} }}^{2} } \\ \end{array} } \right] & ={\mathbf{D}}_{0}/{\left\{ {{\text{tr}}\left[ {{\mathbf{WW}}^{{\prime }} } \right]} \right\}/{\text{n}}} \\ & = \left[ {\begin{array}{*{20}c} {\sigma_{{D_{L} }}^{2} } & {\sigma_{{D_{L} D_{Y} }} } & {\sigma_{{D_{L} D_{LY} }} } \\ {\sigma_{{D_{Y} D_{L} }} } & {\sigma_{{D_{Y} }}^{2} } & {\sigma_{{D_{Y} D_{LY} }} } \\ {\sigma_{{D_{L} D_{LY} }} } & {\sigma_{{D_{Y} D_{LY} }} } & {\sigma_{{D_{LY} }}^{2} } \\ \end{array} } \right]/\left\{ {{\text{tr}}\left[ {{\mathbf{WW}}^{{\prime }} } \right]} \right\}/{\text{n}} \\ \end{aligned}$$

This approach, which is an extension of Vitezica et al. [22], makes it possible to estimate (co)variances of genotypic effects of SNPs in purebred and crossbred populations under a genomic model with additive and non-additive (dominance) inheritance.

Matrices **Z** and **W**, their crossproducts and the inverses of **G** and **D** were built using own programs. Genetic parameters were estimated by using average information REML with software airemlf90 [33]. Standard errors on functions of genetic parameters (i.e. standard errors on correlations) were estimated from the average information matrix using the REML-MVN method of Houle and Meyer [36].

### Additive and dominance variances in purebred and crossbred populations

The additive and dominance (co)variances of genotypic effects of SNPs, either within breed or between breeds, were calculated using Eqs. (6) and (8), respectively. Using these calculated additive and dominance (co)variances of SNPs across all the SNPs, the corresponding traditional, individual-based genetic parameters can be obtained as follows. The genetic parameters obtained are directly comparable to pedigree-based estimates [32].

Consider the allele substitution effect $\alpha = a + \left( {q - p} \right)d$. According to [32], the additive genetic variances for purebred performance (mating animals in the same breed) for breed L ($\sigma_{{AP_{L } }}^{2}$) and Y ($\sigma_{{AP_{Y } }}^{2}$) are:

$$\begin{aligned} \sigma_{{AP_{L } }}^{2} & = \sum \left( {2p_{i}^{L} q_{i}^{L} } \right)\sigma_{{a_{L} }}^{2} + \sum \left( {2p_{i}^{L} q_{i}^{L} \left( {q_{i}^{L} - p_{i}^{L} } \right)^{2} } \right)\sigma_{{d_{L} }}^{2} , \\ \sigma_{{AP_{Y} }}^{2} & = \sum \left( {2p_{i}^{Y} q_{i}^{Y} } \right)\sigma_{{a_{Y} }}^{2} + \sum \left( {2p_{i}^{Y} q_{i}^{Y} \left( {q_{i}^{Y} - p_{i}^{Y} } \right)^{2} } \right)\sigma_{{d_{Y} }}^{2} , \\ \end{aligned}$$

where $\sigma_{a}^{2}$ and $\sigma_{d}^{2}$ are the variances of additive and dominance genotypic effects of SNPs in either breed L or Y; *p*_*i*_ and *q*_*i*_ are allele frequencies for SNP *i*; indices *L* and *Y* denote the breeds Landrace and Yorkshire, respectively. For crossbred performance of say, Landrace, the allele substitution effect is $\alpha_{{AC_{L} }} = a_{{AC_{L} }} + \left( {q^{Y} - p^{Y} } \right)d_{{AC_{L} }}$. Thus, the additive genetic variances within purebred L and Y for crossbred performance (due to gametes from the L or Y individuals in the crossbred population) are equal to:

$$\begin{aligned} \sigma_{{AC_{L} }}^{2} & = \sum \left( {2p_{i}^{L} q_{i}^{L} } \right)\sigma_{{a_{LY} }}^{2} + \sum \left( {2p_{i}^{L} q_{i}^{L} \left( {q_{i}^{Y} - p_{i}^{Y} } \right)^{2} } \right)\sigma_{{d_{LY} }}^{2} , \\ \sigma_{{AC_{Y} }}^{2} & = \sum \left( {2p_{i}^{Y} q_{i}^{Y} } \right)\sigma_{{a_{LY} }}^{2} + \sum \left( {2p_{i}^{Y} q_{i}^{Y} \left( {q_{i}^{L} - p_{i}^{L} } \right)^{2} } \right)\sigma_{{d_{LY} }}^{2} , \\ \end{aligned}$$

where the $\sigma_{{AC_{L} }}^{2}$ represents the additive genetic variance of animals in breed L when mated to animals in breed Y; the $\sigma_{{AC_{Y} }}^{2}$ represents the additive genetic variance of animals in breed Y when mated to animals in breed L; and $\sigma_{{a_{LY} }}^{2}$ and $\sigma_{{d_{LY} }}^{2}$ are the variances of additive and dominance genotypic effects of SNPs in the crossbred LY population, respectively. The additive genetic variance for animals in the crossbred LY population ($\sigma_{{AC_{LY} }}^{2}$) is the sum of the additive genetic variance of Landrace alleles and that of Yorkshire alleles in the crossbred animals [22] as follows:

$$\sigma_{{AC_{LY} }}^{2} = \frac{1}{2}\sigma_{{AC_{L} }}^{2} + \frac{1}{2}\sigma_{{AC_{Y} }}^{2} .$$

Note that this variance is not the additive genetic variance of the crossbred animals acting as reproducers (i.e., creating an F2) [37].

The additive genetic covariances between purebred and crossbred performances within breeds L ($\sigma_{{AP_{L} ,AC_{L} }}$) and Y ($\sigma_{{AP_{Y} ,AC_{Y} }}$) are:

$$\begin{aligned} \sigma_{{AP_{L} ,AC_{L} }} & = \sum \left( {2p_{i}^{L} q_{i}^{L} } \right) \sigma_{{a_{L,LY} }} + \sum \left( {2p_{i}^{L} q_{i}^{L} \left( {q_{i}^{L} - p_{i}^{L} } \right)\left( {q_{i}^{Y} - p_{i}^{Y} } \right)} \right)\sigma_{{d_{L,LY} }} , \\ \sigma_{{AP_{Y} ,AC_{Y} }} & = \sum \left( {2p_{i}^{Y} q_{i}^{Y} } \right) \sigma_{{a_{Y,LY} }} + \sum \left( {2p_{i}^{Y} q_{i}^{Y} \left( {q_{i}^{Y} - p_{i}^{Y} } \right)\left( {q_{i}^{L} - p_{i}^{L} } \right)} \right)\sigma_{{d_{Y,LY} }} , \\ \end{aligned}$$

where $\sigma_{{a_{L,LY} }}$ and $\sigma_{{d_{L,LY} }}$ are the covariances of SNP effects between purebred L and crossbred LY populations for additive and dominance, respectively; $\sigma_{{a_{Y,LY} }}$ and $\sigma_{{d_{Y,LY} }}$ are the covariances of SNP effects between purebred Y and crossbred LY populations for additive and dominance, respectively.

Therefore, the genetic correlations of breeding values between purebred and crossbred performances within L ($r_{{PC_{L} }}$) and Y ($r_{{PC_{Y} }}$) are: $r_{{PC_{L} }} = \frac{{\sigma_{{AP_{L} ,AC_{L} }} }}{{\sqrt {\sigma_{{AP_{L} }}^{2} \sigma_{{AC_{L} }}^{2} } }}$ and $r_{{PC_{Y} }} = \frac{{\sigma_{{AP_{Y} ,AC_{Y} }} }}{{\sqrt {\sigma_{{AP_{Y} }}^{2} \sigma_{{AC_{Y} }}^{2} } }}$.

According to [22], the dominance genetic variances within purebred populations L and Y are $\sigma_{{D_{L} }}^{2} = \sum \left( {2p_{i}^{L} q_{i}^{L} } \right)^{2} \sigma_{{d_{L} }}^{2}$ and $\sigma_{{D_{Y} }}^{2} = \sum \left( {2p_{i}^{Y} q_{i}^{Y} } \right)^{2} \sigma_{{d_{Y} }}^{2}$, respectively. The dominance genetic variance in crossbred LY animals is $\sigma_{{D_{LY} }}^{2} = \sum \left( {4p_{i}^{L} q_{i}^{L} p_{i}^{Y} q_{i}^{Y} } \right)\sigma_{d}^{2}$.

The broad sense heritabilities for purebred performance ($H_{P}^{2}$) were calculated as the ratio of total genetic variances for purebred performance ($\sigma_{AP}^{2} + \sigma_{D}^{2}$) to phenotypic variances ($\sigma_{AP}^{2} + \sigma_{D}^{2} + \sigma_{e}^{2}$).

### Correlations of allele substitution effects between two breeds

The breeding value of an individual includes the allele substitution effects of all genes and the allele frequencies. For purebred performance, the allele substitution effects of one locus for breed L and Y are:

$$\begin{aligned} \alpha_{L} & = a_{L} + \left( {q_{i}^{L} - p_{i}^{L} } \right)d_{L} , \\ \alpha_{Y} & = a_{Y} + \left( {q_{i}^{Y} - p_{i}^{Y} } \right)d_{Y} , \\ \end{aligned}$$

where *a* is the additive effect and *d* is the dominance effect for each SNP; *p*_*i*_ and *q*_*i*_ are allele frequencies for SNP *i*, with superscripts denoting breeds L or Y. In the case of purely additive gene action, the covariance between $\alpha_{L}$ and $\alpha_{Y}$ is $\sigma_{{\alpha_{L,Y} }}$, which can be interpreted as a genetic correlation among populations [38–40]. Then, the covariance between the allele substitution effects of one locus is:

$$\begin{aligned} cov\left( {\alpha_{L} ,\alpha_{Y} } \right) & = cov\left( {a_{L} + \left( {q_{i}^{L} - p_{i}^{L} } \right)d_{L} , a_{Y} + \left( {q_{i}^{Y} - p_{i}^{Y} } \right)d_{Y} } \right) \\ & = cov\left( {a_{L} ,a_{Y} } \right) + \left( {q_{i}^{L} - p_{i}^{L} } \right)\left( {q_{i}^{Y} - p_{i}^{Y} } \right)cov\left( {d_{L} ,d_{Y} } \right) \\ & = \sigma_{{a_{L,Y} }} + \left( {q_{i}^{L} - p_{i}^{L} } \right)\left( {q_{i}^{Y} - p_{i}^{Y} } \right)\sigma_{{d_{L,Y} }} , \\ \end{aligned}$$

where $\sigma_{{a_{L,Y} }}$ and $\sigma_{{d_{L,Y} }}$ are the additive and dominance covariances of SNP effects between breeds L and Y for additive and dominance, respectively. If we assume that SNP effects (both additive and dominance) are independent across loci, then the covariance between the allele substitution effects across all *n* loci is:

$$cov\left( {\alpha_{L} ,\alpha_{Y} } \right) = \sigma_{{\alpha_{L,Y} }} = \sigma_{{a_{L,Y} }} + \frac{1}{n}\sum \left( {\left( {q_{i}^{L} - p_{i}^{L} } \right)\left( {q_{i}^{Y} - p_{i}^{Y} } \right)} \right)\sigma_{{d_{L,Y} }} .$$

Also, the variances of allele substitution effects across all *n* loci for breeds L and Y are:

$$\begin{aligned} var\left( {\alpha_{L} } \right) & = \sigma_{{\alpha_{L} }}^{2} = \sigma_{{a_{L} }}^{2} + \frac{1}{n}\sum \left( {\left( {q_{i}^{L} - p_{i}^{L} } \right)^{2} } \right)\sigma_{{d_{L} }}^{2} , \\ var\left( {\alpha_{Y} } \right) & = \sigma_{{\alpha_{Y} }}^{2} = \sigma_{{a_{Y} }}^{2} + \frac{1}{n}\sum \left( {\left( {q_{i}^{Y} - p_{i}^{Y} } \right)^{2} } \right)\sigma_{{d_{Y} }}^{2} , \\ \end{aligned}$$

where $\sigma_{a}^{2}$ and $\sigma_{d}^{2}$ are the additive and dominance variance of SNPs. Then, the correlation of allele substitution effects for purebred performance between populations L and Y is $r_{{\alpha P_{L} ,\alpha P_{Y} }} = \frac{{\sigma_{{\alpha_{L,Y} }} }}{{\sigma_{{\alpha_{L} }} \sigma_{{\alpha_{Y} }} }}$. If there is no dominance variation, the $r_{{\alpha P_{L} ,\alpha P_{Y} }}$ relates to additive genetic variances as $r_{{\alpha P_{L} ,\alpha P_{Y} }} = \frac{{\sigma_{{a_{L,Y} }} }}{{\sigma_{{a_{L} }} \sigma_{{a_{Y} }} }}$.

The correlation of allele substitution effects for crossbred performance between populations L and Y is similar to that for purebred performance, but the allele frequencies are swapped, as:

$$\begin{aligned} r_{{\alpha C_{L} ,\alpha C_{Y} }} & = \frac{{\sigma_{{\alpha_{L\,in\,LY, Y\,in\,LY} }} }}{{\sigma_{{\alpha_{L\,in\,LY} }} \sigma_{{\alpha_{Y\,in\,LY} }} }} \\ & = \frac{{\sigma_{{a_{LY} }}^{2} + \frac{1}{n}\sum \left( {\left( {q_{i}^{L} - p_{i}^{L} } \right)\left( {q_{i}^{Y} - p_{i}^{Y} } \right)} \right)\sigma_{{d_{LY} }}^{2} }}{{\sqrt {\sigma_{{a_{LY} }}^{2} + \frac{1}{n}\sum \left( {\left( {q_{i}^{Y} - p_{i}^{Y} } \right)^{2} } \right)\sigma_{{d_{LY} }}^{2} } \sqrt {\sigma_{{a_{LY} }}^{2} + \frac{1}{n}\sum \left( {\left( {q_{i}^{L} - p_{i}^{L} } \right)^{2} } \right)\sigma_{{d_{LY} }}^{2} } }}, \\ \end{aligned}$$

where $\sigma_{{a_{LY} }}^{2}$ and $\sigma_{{d_{LY} }}^{2}$ are the additive and dominance variance of SNPs in the crossbred LY population. If there is no dominance variation, the $r_{{\alpha C_{L} , \alpha C_{Y} }}$ is equal to 1, by assumption in the model.

### Scenarios

Variance components, genetic correlations of breeding values between purebred and crossbred performances ($r_{PC}$) within each pure breed and correlations of allele substitution effects for purebred ($r_{{\alpha P_{L} ,\alpha P_{Y} }}$) and crossbred ($r_{{\alpha C_{L} ,\alpha C_{Y} }}$) performance between two pure breeds were first investigated using the full genomic dataset. To explore the effects of using genomic information and the inclusion of dominance deviation on the genetic evaluation of crossbred performance in the trivariate model, three different scenarios were compared.

#### Nogen

The statistical model was a trivariate BLUP model, similar to Eq. (3), but the dominance deviation was excluded. Instead of using a genomic relationship matrix, a single relationship matrix **A** was constructed across the three breeds, assuming that they form a single population. Thus, the genetic (co)variances of additive genetic effects **u** were:

$$Var({\mathbf{u}}) = {\mathbf{A}} \otimes \left[ {\begin{array}{*{20}c} {\sigma_{{A_{L} }}^{2} } & {\sigma_{{A_{L} A_{Y} }} } & {\sigma_{{A_{L} A_{LY} }} } \\ {\sigma_{{A_{Y} A_{L} }} } & {\sigma_{{A_{Y} }}^{2} } & {\sigma_{{A_{Y} A_{LY} }} } \\ {\sigma_{{A_{L} A_{LY} }} } & {\sigma_{{A_{Y} A_{LY} }} } & {\sigma_{{A_{LY} }}^{2} } \\ \end{array} } \right] = {\mathbf{A}} \otimes {\mathbf{A}}_{{\mathbf{0}}} ,$$

where **A**_**0**_ were variance components associated to genetic additive effects and not the genotypic additive effects in Eq. (5). Pedigree-based inbreeding depression was also included in the model. The pedigree-based inbreeding coefficients were calculated as in [41] using the software inbupgf90 [33].

#### Gen_AM

The statistical model was similar to Eq. (3), but without dominance deviations. Genomic information was used to construct the additive genomic relationship matrix.

#### Gen_ADM

The statistical model includes additive and dominance effects as in Eq. (3). Genomic information was used to construct the additive and dominance genomic relationship matrices.

To explore the impact of genomic information and dominance effects on genomic evaluation for crossbred performance, the full genomic dataset was split into training and validation populations and the predictive ability for crossbred animals in the validation population was investigated in different scenarios. The farrowing date of January 1, 2013 was used as the cut-off date to divide recorded purebred and crossbred sows into training and validation populations. As a result, 6769 sows (1270 L, 1405 Y and 4094 LY) were included in the training population, while the remaining 2716 sows (854 L, 813Y and 1049 LY) were included in the validation population. Predictive ability of crossbreds was measured as the correlations $cor\left( {y_{c} ,\hat{y}} \right)$ in the validation population for each scenario, where *y*_*c*_ is the corrected phenotypic records of TNB for crossbred animals; $\hat{y}$ is the predicted corrected observations of TNB for crossbred animals and is equal to the sum of the estimated population mean ($\hat{\mu }$), inbreeding ($f\hat{b}$) and genotypic values ($\hat{g}$); the genotypic value $\hat{g}$ was calculated as the sum of additive and dominance genetic effects in the scenario *Gen_ADM*. In the other two scenarios, the genotypic value $\hat{g}$ only included the additive genetic effect. Hotelling–Williams t test at a confidence level of 5% was applied to evaluate the significance of the differences in validation correlations in each scenario. Furthermore, to detect the possible biases in the predictions, the regression coefficients of *y*_*c*_ on $\hat{y}$ were explored. Note that no bias implies that a regression coefficient equals 1. In addition, to measure the uncertainty associated with the predictions, 1000 bootstrap samples [42] was applied to estimate the means and standard errors.

For comparison, the predictive ability of crossbred animals was also investigated in a model without inbreeding depression effects, for all three scenarios. The predictive ability was measured as the correlation $cor\left( {y_{c} ,\hat{y}} \right)$, where $\hat{y}$ is the sum of the estimated population mean ($\hat{\mu }$) and genotypic value ($\hat{g}$).

## Results

### Variance components, heritabilities and correlations

Table 1 shows the estimates of variance components for additive genetic effects for purebred ($\sigma_{AP}^{2}$) and crossbred ($\sigma_{AC}^{2}$) performance in different scenarios, and dominance variations ($\sigma_{D}^{2}$) in the *Gen_ADM* scenario. For all scenarios, the additive genetic variances for purebred performance ($\sigma_{AP}^{2}$) were larger than those for their crossbred performance ($\sigma_{AC}^{2}$). Estimated variance components in the scenarios *Gen_AM* and *Gen_ADM* were very close, but different from those obtained in scenarios without using genomic information. In general, estimates had large standard errors in all scenarios, but no obvious differences in standard errors were detected between different scenarios. Residual variance for purebred animals ($\sigma_{e}^{2}$) was larger than for crossbred animals ($\sigma_{{e_{LY} }}^{2}$) in each scenario. For the scenario *Gen_ADM*, the ratios of dominance genetic variance to additive genetic variance ranged from 5 to 11% for both purebred and crossbred populations.

**Table 1:** Variance components of additive and dominance genetic effects for purebred and crossbred animals

Scenario	Breed	$\sigma_{AP}^{2}$	$\sigma_{AP,AC}$	$\sigma_{AC}^{2}$	$\sigma_{D}^{2}$	$\sigma_{e}^{2}$	$\sigma_{{AC_{LY} }}^{2}$	$\sigma_{{D_{LY} }}^{2}$	$\sigma_{{e_{LY} }}^{2}$
*Nogen*	L	0.99 (0.31)	0.17 (0.07)	0.05 (0.02)	–	10.82 (0.43)	0.05 (0.02)	–	7.35 (0.15)
	Y	1.07 (0.33)	0.15 (0.07)	0.05 (0.02)	–	8.96 (0.38)			
*Gen_AM*	L	0.87 (0.22)	0.47 (0.10)	0.28 (0.07)	–	10.89 (0.38)	0.28 (0.07)	–	7.11 (0.15)
	Y	0.55 (0.20)	0.17 (0.10)	0.28 (0.07)	–	9.42 (0.33)			
*Gen_ADM*	L	0.86 (0.21)	0.46 (0.10)	0.28 (0.06)	0.04 (0.03)	10.86 (0.38)	0.28 (0.06)	0.02 (0.01)	7.11 (0.15)
	Y	0.54 (0.18)	0.17 (0.09)	0.28 (0.06)	0.06 (0.05)	9.35 (0.33)			

Numbers in brackets are the standard errors of the corresponding parameters

$\sigma_{AP}^{2}$ is the additive genetic variance for purebred performance; $\sigma_{AP,AC}$ is the additive genetic covariance between purebred and crossbred performance; $\sigma_{AC}^{2}$ is the additive genetic variance for crossbred performance; $\sigma_{D}^{2}$ is the dominance genetic variance for either purebred animals; $\sigma_{e}^{2}$ is the residual variance for purebred animals; $\sigma_{{AC_{LY} }}^{2}$ is the additive genetic variance for the F1 crossbred animals LY; $\sigma_{{D_{LY} }}^{2}$ is the dominance genetic variance for the F1 crossbred animals LY; and $\sigma_{{e_{LY} }}^{2}$ is the residual variance for the F1 crossbred animals LY

*L* Landrace, *Y* Yorkshire breeds

The broad sense heritabilities for purebred and crossbred animals, genetic correlations between breeding values for purebred and crossbred performances within pure breeds and correlations of allele substitution effects across the two breeds are in Table 2. In different scenarios, the heritabilities of purebred performance ($H_{P}^{2}$) ranged from 0.07 (0.03) to 0.08 (0.03) and from 0.06 (0.03) to 0.10 (0.03) for breeds L and Y, respectively. Standard errors of $H_{P}^{2}$ were almost consistent across scenarios. Estimated genetic correlations of breeding values between purebred and crossbred performances ($r_{PC}$) increased from 0.76 (0.20) (*Nogen*) to 0.95 (0.06) (*Gen_AM*) for breed L and from 0.43 (0.22) (*Gen_ADM*) to 0.54 (0.30) (*Nogen*) for breed Y. The $r_{PC}$ was higher for breed L than for breed Y in all scenarios, but the standard errors of $r_{PC}$ were always higher for breed Y than for breed L. With genomic information, the correlations of allele substitution effects between purebred ($r_{{\alpha P_{L} ,\alpha P_{Y} }}$) and crossbred ($r_{{\alpha C_{L} ,\alpha C_{Y} }}$) performance between breeds L and Y were estimated, as shown in Table 3. For purebred performance, $r_{{\alpha P_{L} ,\alpha P_{Y} }}$ was equal to 0.14 and 0.19 in *Gen_AM* and *Gen_AM*D, respectively. However, the standard errors were large, around 0.2 in both scenarios. For crossbred performance, $r_{{\alpha C_{L} ,\alpha C_{Y} }}$ was equal to 0.98 in *Gen_ADM*. This high correlation is a byproduct of assuming that additive biological effects in crossbred animals are the same regardless of the Yorkshire or Landrace origin of the allele. However, the same allele has potentially different effects in the respective Landrace or Yorkshire genetic backgrounds, and the difference is modeled through the correlations, hence the low values of $r_{{\alpha P_{L} ,\alpha P_{Y} }}$. Without including the dominance effects in the model *Gen_AM*, $r_{{\alpha C_{L} ,\alpha C_{Y} }}$ was equal to 1 by definition.

**Table 2:** Heritabilities and genetic correlations between breeding values for purebred and crossbred performances

Scenario	Breed	$r_{PC}$	$H_{P}^{2}$
*Nogen*	L	0.76 (0.20)	0.08 (0.03)
	Y	0.54 (0.30)	0.10 (0.03)
*Gen_AM*	L	0.95 (0.06)	0.07 (0.03)
	Y	0.44 (0.20)	0.06 (0.03)
*Gen_ADM*	L	0.93 (0.05)	0.08 (0.03)
	Y	0.43 (0.22)	0.06 (0.03)

Numbers between brackets are the standard errors of the corresponding parameters

$r_{PC}$ is the genetic correlation of breeding values between purebred and crossbred performances within the Landrace or Yorkshire breeds; $H_{P}^{2}$ is the broad sense heritability for purebred performance for the Landrace and Yorkshire breeds in different scenarios

*L* Landrace, *Y* Yorkshire

**Table 3:** Correlations of allele substitution effects for purebred and crossbred performance between Landrace and Yorkshire breeds

Scenario	$r_{{\alpha P_{L} ,\alpha P_{Y} }}$	$r_{{\alpha C_{L} ,\alpha C_{Y} }}$
*Nogen*	–	–
*Gen_AM*	0.14 (0.22)	1
*Gen_ADM*	0.19 (0.24)	0.98 (0.02)

Numbers between brackets are the standard errors of the corresponding parameters

$r_{{\alpha P_{L} ,\alpha P_{Y} }}$ is the correlation of allele substitution effects for purebred performance between the Landrace and Yorkshire breeds; $r_{{\alpha C_{L} , \alpha C_{Y} }}$ is the correlation of allele substitution effects for crossbred performance between the Landrace and Yorkshire breeds. For *Gen_AM*, $r_{{\alpha C_{L} , \alpha C_{Y} }}$ is equal to 1 by definition

### Predictive abilities

Predictive abilities for crossbred pigs in the validation population are in Table 4. The correlation between the corrected phenotypic values and the predicted observations for TNB ($cor\left( {y_{c} ,\hat{y}} \right)$) ranged from 0.010 in the scenario *Nogen* to 0.056 in scenarios *Gen_AM* and *Gen_ADM*. Standard errors of $cor\left( {y_{c} ,\hat{y}} \right)$ based on 1000 bootstrap samples were equal to 0.03 across all scenarios. No significant differences in predictive ability between scenarios were detected by the Hotelling–Williams t test at the confidence level of 5%.

**Table 4:** Predictive ability for crossbred animals in the validation population

	*Nogen*	*Gen_AM*	*Gen_ADM*
$cor\left( {y_{c} ,\hat{y}} \right)$ ^a^	0.010 (0.031)	0.056 (0.031)	0.056 (0.031)
Regression coefficient^b^	0.703 (2.218)	0.736(0.386)	0.730 (0.385)

Numbers between brackets are the standard errors of the corresponding parameters

^a^Predictive ability (${\text{cor}}\left( {{\text{y}}_{\text{c}} ,\hat{y}} \right)$) is given by the correlation coefficient between the corrected phenotypes ($y_{c}$) and their predictions ($\hat{y}$) for total number of piglets born (TNB) in crossbred animals

^b^Regression coefficient of the corrected phenotypes ($y_{c}$) on the predicted observations ($\hat{y}$) in crossbred animals

The regression coefficients of corrected phenotypic values on the predicted corrected observations for TNB are in the second row of Table 4. Regression coefficients were smaller than 1 for the three scenarios. Among these scenarios, regression coefficients for scenarios with genomic information (*Gen_AM* and *Gen_ADM*) were slightly closer to 1 than that for the pedigree-based scenario (*Nogen*). Except for the *Nogen* scenario, standard errors of regression coefficients were around 0.39. For the *Nogen* scenario, the standard error was around 5 times larger than that for other scenarios. Overall, there was no clear trend towards a scenario with less bias.

For comparison, predictive abilities $cor\left( {y_{c} ,\hat{y}} \right)$ for crossbred pigs in the validation population for the models without the inbreeding depression effect were equal to −0.08 in scenario *Nogen*, 0.045 in scenario *Gen_AM* and 0.046 in scenario *Gen_ADM*. In all cases, these are lower than the predictive abilities in Table 4, and these differences are statistically significant according to the Hotelling–Williams t test.

### Inbreeding depression

Marker-based and pedigree-based inbreeding coefficient (*f*) for each population and their estimated corresponding inbreeding depression parameters (*b*) in the different scenarios are in Table 5. Marker-based inbreeding coefficients were almost identical for breeds L and Y, but they were larger than those for LY, which was expected because crossbred animals have a higher level of heterozygozity than purebred animals. However, according to the pedigree-based inbreeding coefficients, the Landrace population was slightly more inbred than the Yorkshire population. In terms of inbreeding depression parameters (*b*), they were all negative (thus, genomic inbreeding has detrimental effects for TNB even in crossbred animals) but not of the same magnitude across the three populations. Note that for the scenario *Nogen*, *b* was estimated based on the pedigree-based inbreeding coefficients. As a whole, breed L had the most negative *b*, while breed Y had the least negative *b*, regardless of the scenario. Thus, TNB was more negatively affected by inbreeding in breed L than in breed Y and population LY.

**Table 5:** Marker-based and pedigree-based inbreeding coefficients ***f*** and estimated inbreeding depression parameter *b* (piglets per 100% of inbreeding) in different scenarios for each breed

	L	Y	LY
Marker-based inbreeding coefficient *f* ^a^	0.695 (0.019)	0.698 (0.020)	0.565 (0.012)
Pedigree-based inbreeding coefficient *f* ^b^	0.111 (0.032)	0.078 (0.031)	0
*Nogen* (*b*)	−4.821	−3.561	0
*Gen_AM* (*b*)	−9.656	−1.924	−5.122
*Gen_ADM* (*b*)	−9.731	−1.878	−5.055

The inbreeding coefficient is the mean inbreeding coefficient across individuals within each breed

Numbers between brackets are the standard deviations of the mean inbreeding coefficient

For *Nogen*, the inbreeding depression parameter *b* is the regression of phenotype on pedigree-based inbreeding. For *Gen_AM* and *Gen_ADM*, the inbreeding depression parameter *b* is the regression of phenotype on marker-based inbreeding

^a^Calculated as the proportion of homozygous loci per individual

^b^Calculated as in Meuwissen and Luo [41]

## Discussion

This study extended the trivariate GBLUP model of Vitezica et al. [22] in order to obtain (co)variances of effects of SNPs, genetic correlations of breeding values between purebred and crossbred performances and correlations of allele substitution effects under dominance. We also evaluated this model using different scenarios for the genetic evaluation of crossbred performance in Danish purebred and crossbred pigs. Scenarios that included or not genomic information were studied to estimate the genetic correlations of breeding values between purebred and crossbred performances. To our knowledge, this is the first study to report correlations of allele substitution effects between two breeds in the presence of dominance effects. The results show that the Vitezica model [22] is a tool that can be used for the genomic evaluation of crossbred performance in genotyped animals. In this study, for TNB, models with dominance deviations did not improve the genomic evaluation of crossbred performance with regard to both predictive ability and unbiasedness, but the inclusion of an inbreeding depression effect in the models significantly improved predictive ability.

Phenotypic variances were larger for purebred animals (11.76 for breed L and 9.99 for breed Y) than for crossbred animals (7.30 for LY). This could be the reason why the estimated additive genetic variances for purebred performance ($\sigma_{AP}^{2}$) were larger than those for crossbred performance ($\sigma_{AC}^{2}$). However, compared to results in a previous study that used a much larger Danish purebred and crossbred dataset [17], both estimated additive genetic variances and phenotypic variances in the current study were smaller, which is due to three reasons. (1) The dataset in the current study was a genotyped subset of the population used in the previous study. Purebred genotyped individuals were pre-selected and their performances were more homogeneous than that of the whole population. The preselection process resulted in a loss of about 15% of the purebred phenotypic variation. However, the genotyped crossbred animals were an almost random sample of the whole population and there was only a small loss of about 5% of phenotypic variation for crossbred animals. (2) The phenotypic values for TNB in the current study were pre-corrected for fixed and non-genetic random effects. This pre-correction led to a loss of about 11 and 17% of phenotypic variation for purebreds and crossbreds, respectively. (3) During the pre-correction, some genetic variation may have been allocated to other random effects (e.g. service boar effects), in particular because TNB is a lowly heritable trait.

The estimated heritabilities of TNB for purebred performance ($H_{P}^{2}$) were slightly lower than those previously reported (0.11 and 0.09 for breeds L and Y, respectively) [17, 22, 43]. Large standard errors of $H_{P}^{2}$ implied that the current dataset was not large enough. The consistent standard errors across scenarios indicated that even when genomic information was included, the uncertainty of $H_{P}^{2}$ did not decrease. Taking the standard errors into account, the estimated $H_{P}^{2}$ across scenarios were not very different. Compared to the results of [17], the lower $H_{P}^{2}$ found in the current study was due to the sharp decrease in additive genetic variances ($\sigma_{AP}^{2}$).

The ratios of estimated dominance genetic variances to additive genetic variances in the current study (5 to 11%) were generally a little smaller than in other studies on TNB. Vitezica et al. [22] reported that this ratio was equal to about 20% for litter size in both purebred and crossbred lines by using the same trivariate GBLUP model. Esfandyari et al. [19] stated that, by using purebred genomic information in a univariate Bayesian mixture model at the SNP level, the ratio between dominance variance and additive variance for TNB was equal to 15 and 18% for breeds L and Y, respectively. Based on pedigree information, Misztal et al. [10] reported a ratio that reached about 25% for number of piglets born alive in a Yorkshire population. However, there are some studies that did report smaller ratios than those reported here. For instance, Hidalgo [20] reported that, based on genotyped crossbred animals, the dominance variance for TNB accounted for nearly zero of the total genetic variance and concluded that TNB was not affected by dominance effects in the Dutch Landrace and Yorkshire populations. For other traits or species, different ratios of dominance genetic variance to additive genetic variance were also reported. For average daily gain in Duroc pigs, Su et al. [9] estimated a ratio of 15%, but their results were based on genotypic variance components and cannot be directly compared to genetic variance components [32]. For average daily weight gain in Yorkshire and Landrace pigs, Lopes et al. [44] reported ratios of 13.8 and 28%, respectively by including genomic information. For Fleckvieh cattle, Ertl et al. [12] calculated ratios that ranged from 3.4% for stature to 69% for protein yield by using a univariate SNP-BLUP model. Overall, these different ratios of dominance genetic variance to additive genetic variance may reflect differences in the traits analyzed and in the type of information used for the estimation [9], and also uncertainty in the estimates.

The genetic correlation of breeding values between purebred and crossbred performances ($r_{PC}$) is a key parameter in crossbreeding schemes [2]. In the current study, the estimated $r_{PC}$ was in line with results reviewed by Wei et al. [3]. Lutaaya et al. [5] also reported $r_{PC}$ that ranged from 0.32 to 1. Such differences in $r_{PC}$ may reflect differences in the extent of GxE interactions and the distance across breeds. In our study, estimated $r_{PC}$ did not vary dramatically across the scenarios, when the standard errors were taken into account. These standard errors were very large, which indicated that the amount of available information was too small to ensure accurate $r_{PC}$ estimates. Across scenarios, standard errors of $r_{PC}$ decreased when genomic information was included, which indicates that including genomic information may reduce the uncertainty of the estimations. $r_{PC}$ was larger for breed L than for breed Y, which was in agreement with a previous study [17] and may be due to the data structure. Among the 5143 crossbred animals, the number of Yorkshire sires (N = 1125) was much smaller than that of Landrace sires (N = 4018). Such a different amount of information affects the accuracy of the estimates, and thus the standard error of $r_{PC}$ was larger for breed Y than for breed L (see Table 2). However, compared to the results reported in [17], the $r_{PC}$ for breed L increased by about 10% while that for breed Y did not change much. Both pre-correction of data and the genotyped subset of original data used may play a role in the differences observed between the current and previous results [17]. In the previous study, a single-step method, which can use pedigree information and genomic information simultaneously, was used. In this study, the use of only phenotypic records on genotyped individuals affected the accuracy of estimates. Our results confirmed the moderate value of the $r_{PC}$ for TNB in breeds L and Y.

To our knowledge, this is the first time that correlations of allele substitution effects for both purebred ($r_{{\alpha P_{L} ,\alpha P_{Y} }}$) and crossbred ($r_{{\alpha C_{L} ,\alpha C_{Y} }}$) performance between two breeds in the presence of dominance variation are estimated. In genomic selection, SNPs are assumed to be in LD with QTL along the whole genome [45]. The correlation of allele substitution effects between breeds measures the degree of average similarities between SNP effects assuming that the QTL effects are the same in breeds 1 and 2 [38–40]. In practice, the correlation of allele substitution effects between two breeds can be interpreted as indicating “how consistent the SNP substitution effects are across two breeds”. For purebred performance, the estimated SNP substitution effects were based on the within-breed allele frequencies. A high $r_{{\alpha P_{L} ,\alpha P_{Y} }}$ correlation means that the estimated SNP substitution effects based on allele frequencies from breed L can be used for breed Y and vice versa. However, $r_{{\alpha P_{L} ,\alpha P_{Y} }}$ was not significantly different from 0 in the current study, which demonstrates that SNP effects estimated from a reference population that consists of one pure breed (e.g. Landrace) cannot be readily applied to the other breed (e.g. Yorkshire). This was in agreement with the findings of [46] who reported that prediction based on an across-population reference panel was worse than within-population prediction. In other species, estimated correlations of allele substitution effects between breeds based on models without dominance, oscillate between 0 and 0.8, and are trait-dependent [38, 47]. For crossbred performance, an $r_{{\alpha C_{L} ,\alpha C_{Y} }}$ close to 1 was found in the current study, which indicated that the allele substitution effects based on the allele frequencies from the opposite breeds were very similar for the L and Y breeds. In practice, this suggests that SNP substitution effects that are estimated based on a reference population consisting of crossbred animals can be used to estimate crossbred breeding values for both breeds L and Y.

It was expected that genomic evaluations obtained by including dominance deviations in the model would be improved, especially when records of crossbred animals were included [9]. However, our results showed that inclusion of dominance deviations did not increase the predictive ability for crossbreds. This result was in line with conclusions in [9, 12, 20], but was opposite to those in [18, 19, 48, 49]. Theoretically, estimating dominance genetic effects should be useful because ignoring them will result in less accurate estimates of allele substitution effects and consequently less accurate estimated breeding values in genomic prediction [11]. However, regarding the additive genetic variance, estimates were nearly the same in scenarios *Gen_AM* and *Gen_ADM*, which demonstrated that the additive variances were already well captured by the additive model. Thus, the accuracy of the estimated additive genetic effects was not affected when dominance effects were included in the model [12]. Moreover, a simulation study at the level of the gene action showed that when all gene actions were purely additive, including dominance in addition to the additive effects in the model was not advantageous compared to using an additive model. Hidalgo [20] showed that TNB was not affected by dominance in the Dutch crossbred population. In the current study, we also observed similar results, and dominance variation accounted for a small proportion of the total genetic variation (4 to 10%). The lack of change in predictive ability also indicated the difficulty of distinguishing dominance genetic effects from additive genetic effects [9], but it confirmed a previous simulation study that concluded that the use of a dominance model did not negatively affect genomic evaluation even if the trait was purely additive [18].

Scenarios in which genomic information was included (*Gen_AM* and *Gen_ADM*) showed higher predictive abilities than the pedigree-based scenario (*Nogen*). For the *Nogen* scenario, the relationship matrix was constructed based on a base population that was considered as a mixture of L and Y animals, which was not the case. Therefore, the results of the *Gen_AM* and *Gen_ADM* scenarios were more reliable than those of the *Nogen* scenario. Although predictive abilities were not significantly different according to the Hotelling-Williams t-test, the results from 1000 bootstrap samples still showed that the predictive abilities of about 90% of the crossbred animals would be higher when genomic information was available (894 of 1000 bootstrap samples showed higher predictive abilities in scenarios that included genomic information than those in the *Nogen* scenario; results not shown). Comparison of the predictive abilities that were estimated in the current study with those from a previous study [17] indicated that the single-step model [16] might be more robust than the Vitezica model [22] used in this paper in terms of both predictive ability and unbiasedness for the crossbred performance. Our results suggested that using a small set of genotyped animals and pre-corrected data to implement genetic evaluation for crossbred performance was less powerful than using the whole dataset, which is similar to the conclusions for purebred performance [43].

The regression coefficients obtained with the Vitezica model were less than 1, which suggests that variations in total genetic effects could be overestimated (inflated). In terms of unbiasedness, there was no clear trend among the scenarios examined, regardless of whether genomic information was included or not. Overall, unbiasedness was not a problem in the current study because the regression coefficients in all scenarios did not significantly differ from 1.

Inbreeding depression for litter size in pigs is a well-known phenomenon [50, 51], and we found that inclusion of inbreeding effects in the model improved predictive abilities of crossbred animals. Estimates of inbreeding depression effects are rarely reported, but our estimates agree with those previously reported for commercial and Iberian pigs [52]. Inbreeding depression was, for the same amount of marker-based inbreeding, more detrimental in the Landrace than in the Yorkshire breed. There are many possible explanations among which the purging of lethal recessive alleles [53]. We also report an estimate of the inbreeding depression parameter for the crossbred animals, which is between the estimates for the parental breeds. To our knowledge, this estimate has never been reported.

The correlation between breeding values and dominance deviations is of theoretical concern [30]. However, this does not apply to the current marker-based analyses for the following reasons. (1) In a pedigree-based analysis, mating in an inbred population produces deviations from the Hardy–Weinberg equilibrium, which generate correlations between breeding values and dominance deviations [30]. However, in our study, SNPs are in Hardy–Weinberg equilibrium if allele frequencies are considered in the current generation. (2) Such a correlation occurs because the pedigree information forces the genetic model to refer to the base population, since the state of alleles is not known, i.e. only probabilities of IBD are known. In our study, the states of alleles are known and the model can be described as referring to the current generation instead. (3) The equivalent GBLUP models in Eq. (3) used genotypic additive and dominance values, not breeding values and dominance deviations. A reasonable assumption in the model is that additive and dominance effects are unrelated at each SNP. Thus, covariance between additive and dominance genetic effects was ignored in the current study.

## Conclusions

We present for the first time the use of genomic inbreeding in crossbred and purebred genomic evaluation. Estimates are biologically sound and are relevant even for crossbred animals. We also report for the first time, estimated correlations of allele substitution effects in the presence of dominance. For TNB, the dominance genetic variance accounts for only a small proportion of the total genetic variation (4 to 10%). A moderate, positive genetic correlation between breeding values for TNB for purebred and crossbred performances was confirmed. Inclusion of dominance in the GBLUP model did not improve predictive ability for crossbred animals, whereas inclusion of inbreeding depression effects did. An additive GBLUP model is sufficient to capture the additive genetic variances and for genomic evaluation. The GBLUP model [22] was applied successfully for genetic evaluations for crossbred performance in pigs. This model can potentially be a useful tool in genetic evaluation for crossbred performance.
